# The emerging role of insulin-like growth factors in testis development and function

**DOI:** 10.1186/2051-4190-24-12

**Published:** 2014-08-18

**Authors:** Richard J Griffeth, Vanessa Bianda, Serge Nef

**Affiliations:** Department of Genetic Medicine and Development, University of Geneva Medical School, 1211 Geneva 4, Switzerland; iGE3, Institute of Genetics and Genomics of Geneva, University of Geneva, Geneva, Switzerland

**Keywords:** IGF-I, IGF-II, Insulin, Sertoli cells, Leydig cells, Spermatogenesis, Sex determination, Testis, IGF-I, IGF-II, Insuline, Cellules de Sertoli, Cellules de Leydig, Spermatogenèse, Détermination sexuelle, Testicule

## Abstract

The insulin-like family of growth factors (IGFs) – composed of insulin, and insulin-like growth factors I (IGF1) and II (IGF2) – provides essential signals for the control of testis development and function. In the testis, IGFs act in an autocrine-paracrine manner but the extent of their actions has been underestimated due to redundancies at both the ligand and receptor levels, and the perinatal lethality of constitutive knockout mice. This review synthesizes the current understanding of how the IGF system regulates biological processes such as primary sex determination, testis development, spermatogenesis and steroidogenesis, and highlights the questions that remain to be explored.

## Résumé

La famille des facteurs de croissance de type insuline, composée de l'insuline et des deux facteurs de croissance ressemblant à l'insuline que sont l'IGF-I et l'IGF-II, est essentielle pour le contrôle du développement et de la fonction testiculaire. Ces facteurs agissent de manière autocrine et paracrine entre les différents types de cellules germinales et somatiques qui composent le testicule. Cependant, leur étude dans le contexte testiculaire reste complexe et l'étendue de leurs actions est généralement sous-estimée, notamment du fait de redondances fonctionnelles tant au niveau des ligands que de leurs récepteurs, mais également en raison de la létalité périnatale observée chez les souris invalidées pour ces gènes. Le but de cette revue est d'offrir à la fois une synthèse des connaissances sur la fonction des IGFs au cours de la détermination du sexe, du développement testiculaire, de la spermatogenèse et de la stéroïdogenèse, mais aussi d’évoquer les axes de recherches en cours et de mettre en lumière les questions qui restent à explorer.

## Introduction

Male fertility depends on proper testis function. The testis is specified in the fetus from a bipotential gonad and its development follows an intricate course of genetic, hormonal, and growth factor expression. When properly executed this process leads to normal testicular function, which includes the production of mature sperm and testosterone. Spermatogenesis is a complex biological process that involves the proliferation and differentiation of diploid spermatogonia into mature haploid spermatozoa capable of fertilization. Testosterone production relies on the endocrine action of hormones released from the brain and testosterone acts locally in the testis to stimulate spermatogenesis. In addition, autocrine-paracrine signaling of growth factors in the testis is essential for proper testis function. Thus, male fertility, testis function and spermatogenesis are regulated by a complex interplay of autocrine, paracrine, and endocrine factors.

The insulin family of growth factors (insulin, insulin-like growth factors I (IGF1) and II (IGF2) are small single-chain mitogenic polypeptides that provide essential signals for the control of growth, metabolism and reproductive functions [1, 2]. In particular, the insulin/IGF system plays a pivotal role in the regulation of cell growth, proliferation, differentiation and survival and affects nearly every organ and system in the body [3, 4]. Importantly, it also plays a major role in the proper development and function of the testis. Although reproductive capability is regulated by the hypothalamic-pituitary-gonadal axis [5], the activity of local gonadal factors, such as those of the insulin/IGF family, modulate reproductive performance. For example, *Igf1* null males are infertile dwarfs and exhibit a reduction of greater than 80% in both spermatogenesis and serum testosterone levels [6]. This not only highlights the overall importance of IGF signaling for body growth and development, but also emphasizes the critical role of IGFs in reproductive function.

More recent studies utilizing mouse functional genetics and the *Cre/Lox* system have evaluated the role of some of the receptors, ligands, and signaling molecules of the insulin/IGF signaling pathway and have provided more detailed information that underscores its indispensable role in various aspects of testicular development and function [7–10]. However, the precise roles of IGFs in various facets of testicular development, spermatogenesis and steroidogenesis are still unknown. Our relatively limited understanding arises from several complexities, including redundancies between IGFs, their receptors, and their intracellular modulators; the embryonic or early postnatal lethality of mutant animals; and the complex intratesticular autocrine-paracrine regulation. Recent data have suggested that certain cell types within the testis may be a source of IGF production and that the synthesis of these local IGFs may function via autocrine-paracrine action within the testis to regulate various aspects of testicular development and function.

The purpose of this review is to synthesize the available data derived from studies in both laboratory animals and humans regarding the role of the insulin family of growth factors in male reproductive development and function. To provide perspective, we begin with a brief overview of the players involved and their biological actions. We then describe the essential roles of IGFs during sex determination and testicular development, and how they modulate the function of Sertoli cells (SCs), germ cells (GCs), and Leydig cells (LCs). Finally, we draw conclusions from the current data and provide insight into unresolved issues and future experimentation.

### The IGF family and their receptors

**Figure 1:**
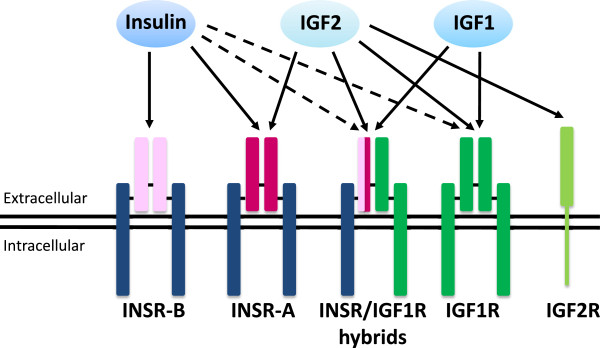
**Receptors for insulin, IGF1 and IGF2.** INSR and IGF1R are composed of two αβ dimers which associate to form heterotetrameric complexes. The αβ dimers are linked together by disulfide bonds and two dimers are also linked by disulfide bonds to form the tetramer. The α subunit is the extracellular portion of the receptor while the β subunit spans the membrane and its cytoplasmic portion interacts with IRS proteins which are key intracellular mediators of insulin/IGF signaling. Single αβ dimers are derived from separate genes and the INSR has two splice variants, INSR-B and INSR-A. Each variant shares the same membrane-spanning β subunit (dark blue) but differs in the extracellular α subunit (light pink or dark pink, respectively). The IGF1R has different α and β subunits compared to the INSR (dark green). These combinations of αβ dimers allow for hybrid receptors, which bind insulin, IGF1, and IGF2 with differing affinities. The schematic shows the αβ dimers, α2β2 hybrid receptors, and the known ligands that bind each receptor. Relative binding affinities are represented by arrows, where a solid arrow signifies a higher binding affinity than a broken arrow.

Insulin, IGF1 and IGF2 modulate a variety of cellular activities including cell survival, proliferation, differentiation and metabolism [3, 4]. The physiological effects of these factors are mediated through the activation of two related tyrosine kinase receptors: the insulin receptor (INSR), and the type-I insulin-like growth factor receptor (IGF1R). INSR and IGF1R are heterotetrameric glycoproteins composed of two extracellular α subunits and two transmembrane β subunits linked together by disulfide bridges (see Figure 1 and [11]).

The complexity of insulin/IGF signaling results from the multiplicity of ligands (insulin, IGF1, IGF2), receptors (INSR, IGF1R), IGF binding proteins (IGFBP1 to 6), and IGFBP proteases, as well as complex downstream signaling pathways. Although they provide one source of complexity, the INSR and IGF1R themselves are quite similar, sharing 84% amino acid similarity in the β subunit cytoplasmic region, where the tyrosine-specific kinase domain is found, 64-67% similarity in the extracellular α subunit region [12], and 100% similarity in the ATP binding domain. Interestingly, additional complexity arises from the existence of hybrid INSR/IGF1R receptors. Both INSR and IGF1R are the products of single genes, with two subunits being derived from a single chain precursor to give each αβ subunit complex. Two αβ dimers are linked with disulfide bonds to form the resulting tetramer. However, when αβ dimers from each gene are linked with disulfide bonds, hybrid INSR-IGF1R complexes are formed, which serve as an additional receptor isoform. A third layer of complexity results from differential mRNA splicing of the INSR α subunit into two isoforms, which increases the number of potential receptors. INSR-A lacks exon 11, which encodes 12 amino acids near the C-terminus of the α subunit in INSR-B [13]. As a result of these factors, there are five potential receptors from the two receptor genes. Finally, variable binding affinities add another level of intricacy to insulin/IGF signaling. The affinity of INSR-A and INSR-B isoforms and IGF1R towards each insulin/IGF ligand differs significantly (Figure 1). Insulin binds with high affinity to INSR-A and INSR-B but also binds with a lower affinity to IGF1R and the hybrid receptors INSR-A/IGF1R and INSR-B/IGF1R [14, 15]. IGF1 preferentially binds to IGF1R and the hybrid receptors, but also binds to INSR-A and INSR-B with a lower affinity [16–20]. IGF2 binds to IGF1R and INSR-A with an affinity close to that of insulin, and also binds to INSR-A/IGF1R and INSR-B/IGF1R albeit with lower affinity [14]. IGF2 also binds to the cation-independent mannose-6-phosphate/IGF2 receptor (M6P/IGF2R), but this receptor lacks intrinsic tyrosine kinase activity and probably serves as a mechanism to clear circulating IGF2 [21].

**Figure 2:**
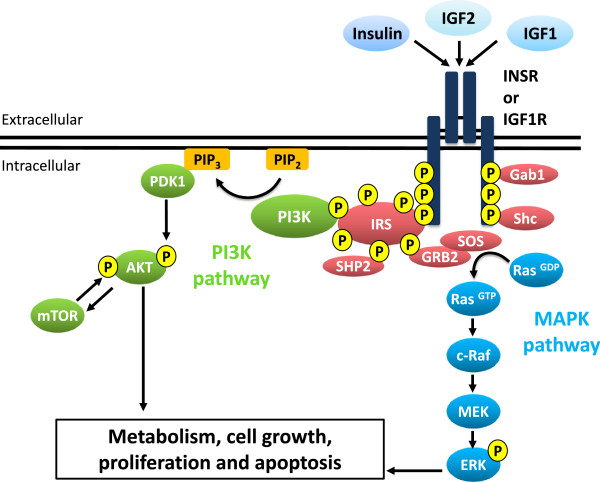
**A simplified view of insulin/IGF1 signaling.** Insulin/IGF1 signaling is mediated by a complex, highly integrated network that controls several processes. Two major pathways are activated by insulin/IGF1 signaling, the ATK/PI3K pathway and the ERK/MAPK pathway, which are involved in several cellular processes such as metabolism, cell growth, proliferation, and apoptosis. Activation of the INSR/IGF1R by insulin/IGF1 binding leads to autophosphorylation of the β subunits and the receptor tyrosine kinase subsequently phosphorylates IRS proteins on their tyrosine residues. This creates recognition sites for additional effector molecules containing SH2 domains, such the p85 regulatory subunit of PI3K (which activates the AKT/PI3K pathway and is mainly responsible for the metabolic actions of insulin/IGF1) and GRB2 (which activates the ERK/MAPK pathway and primarily regulates cell growth and differentiation). Additionally, the INSR and IGF1R can phosphorylate other substrates, such as SHC and GAB1, which link multiple pathways. Together, these signals stimulate a variety of different downstream biological effects including mitogenesis, gene expression, glucose transport, and glycogen synthesis.

Transduction of signals initiated by ligands in the insulin/IGF signaling pathway is mediated by a complex, highly integrated network that activates several processes. Figure 2 briefly illustrates these signaling pathways. IGF ligands bind to the extracellular α subunits, transmitting a signal across the plasma membrane that activates the intracellular tyrosine kinase domain of the β subunit. The receptor then undergoes a series of phosphorylation reactions in which one β subunit in the heterotetramer phosphorylates specific tyrosine residues on its adjacent β subunit [22]. Interestingly, unlike other receptor tyrosine kinases, which bind directly to the cytoplasmic tails of downstream effectors, the INSR and the IGF1R utilize insulin receptor substrate (IRS) proteins to mediate the binding of intracellular effectors [23]. Four IRS proteins, named IRS1-4, have been identified [24] and are linked to the activation of two major signaling pathways: phosphatidyl-inositol 3-kinase (PI3K) and mitogen activated protein kinase (MAPK), both of which are associated with proliferation, differentiation, metabolism, and cell survival [24, 25]. Nevertheless, it is becoming increasing clear that IGF1 signals via other pathways such as the Janus Kinase/Signal Transducer and Activator of Transcription (JAK-STAT) pathway, which provides further intracellular crosstalk and cell-specific effects of IGF1 [26]. Whether this signaling pathway is important for testicular function and development remains to be determined, however the presence of STAT proteins in structural components of human sperm suggests that their role is different from their transcription factor activity in somatic cells [27]. A further level of insulin/IGF1signaling complexity has been revealed by recent studies in various cells and tissues that indicate INSR and IGF1R are able to translocate to the cell nucleus and function like transcription factors by binding to specific promoters and enhancers to control gene expression (for review see [28]). To date, no such studies have been performed in the testis but genomic effects of INSR and IGF1R would provide yet another layer of complexity to insulin/IGF1 signaling and the challenges facing researchers in the future.

### Biological effects of insulin and IGFs

IGF1 and IGF2 are single chain polypeptides, with 62% sequence identity to proinsulin [11] and are produced by almost every cell in the body, unlike insulin which is specifically expressed in the pancreatic β cells of the islets of Langerhans. The availability of IGFs is modulated by at least six high-affinity binding proteins, which increase the half-life of IGF in serum, prevent the overstimulation of cell growth or excessive apoptosis [29], and regulate the transport of IGFs and their interactions with receptors [30]. The IGF system is part of a major growth-promoting signaling system [3] involved in embryonic and postnatal development. While IGF1 has continuous function throughout development [18, 19], IGF2 is essential only for normal embryonic growth [31, 32]. Since the PI3K and MAPK pathways are involved in a plethora of cellular activities, insulin/IGF signaling regulates and/or is associated with a multitude of functions. Alterations in the insulin/IGF signaling system are associated with significant pathologies including diabetes and cancer, along with dwarfism, dementia, decreased longevity, and various metabolic diseases [33].

### Insulin

Insulin is the most potent of the known anabolic hormones, and is essential for appropriate tissue development, growth, and maintenance of whole-body glucose homeostasis [34]. Insulin is secreted by the β cells of the pancreatic islets of Langerhans in response to increased circulating levels of glucose, and regulates glucose homeostasis by reducing hepatic glucose output via decreased gluconeogenesis and glycogenolysis and increasing the rate of glucose uptake into muscle and adipose tissue [35]. Insulin also profoundly affects lipid metabolism, increasing lipid synthesis in liver and fat cells and attenuating fatty acid release from triglycerides in muscle and adipose tissue [22]. It also plays an indirect role in the regulation of fetal growth by modulating the expression of IGFs, and has direct effects on adipose tissue and the proliferation of the cells within the fetus [4]. A striking feature of insulin expression is that it is almost completely restricted to pancreatic β cells and its effects are therefore mediated in an endocrine manner. Despite a few publications suggesting a link between insulin and testis development and function [36, 37], there is no direct evidence to confirm that insulin plays a role in this process.

### IGF1

IGF1 is primarily produced in the liver but is also synthesized by almost all tissues in the body [38], and plays a significant role in overall growth and adult body size, while also sustaining postnatal development, including reproductive functions [18, 39]. Local production of IGF1 plays a major role in the growth of tissues [40], and due to the overall pattern of IGF production during development, is considered to be more important for postnatal growth and development than insulin or IGF2 [11]. Results from studies in humans with deficiencies in IGF1 or IGF1R are consistent with those in mice, and indicate that normal IGF1 and IGF1R activity is required for proper growth and development. The clinical phenotype of individuals with homozygous IGF1 defects consists of severely reduced pre- an postnatal growth, extreme microcephaly, sensorineural deafness, failure to thrive, and poor feeding during infancy and early childhood, while those with heterogeneous defects in IGF1 or IGF1R show a similar but less severe phenotype [41–43].

Immunostaining in the human testis has shown that IGF1 is preferentially expressed in SCs, with reduced expression found in primary spermatocytes and LCs. IGF1R on the other hand is highly expressed in secondary spermatocytes and early spermatids, with reduced expression in SCs and LCs [44]. While *Igf1* null male mice exhibit intrauterine growth retardation, postnatal growth failure and infertility characterized by a severe decline in spermatogenesis and testosterone production [6], this study also highlights the difficulties in delineating the numerous roles of IGF1 in the testis with a constitutive knockout.

### IGF2

IGF2 is a single polypeptide, like IGF1, with which it also shares 70% of its amino acid sequence [11]. The *Igf2* gene is part of a cluster of imprinted genes [45], and is therefore only produced from the paternal allele, the maternal allele being transcriptionally silent [32, 46]. IGF2 plays a fundamental role in embryonic and fetal development, whereas its role in postnatal development appears to be less significant, as it can largely be replaced by IGF1. IGF2 regulates trophoblast development and function at the feto-maternal interface and is an important factor in the determination of body size. Deletion of *Igf2* results in placental insufficiency and reduced fetal weight [31, 47], whereas its overexpression leads to increased size [48]. Most defects reported in *Igf2* null mice are associated with intrauterine growth restriction [49, 50] and while no testicular defects have been described in *Igf2*-deficient mice, this does not preclude a putative role of IGF2 in testis development and function. The inherent redundancies in the IGF signaling system increase the difficulties in ascertaining the role of IGF2 in testicular function, as the biological actions of IGF2 may be masked by other IGFs. Stage-specific developmental studies in testis cell-specific conditional *Igf2* knockout models may help clarify these issues.

Taken together, these data highlight the importance of the insulin family of growth factors in reproduction, growth, and development. However the use of constitutive KO models leads to a multitude of effects in multiple cell types, making it difficult to draw decisive conclusions about which cell types in the testis are specifically affected. In addition, many constitutive KO models of the insulin family of growth factors result in embryonic or early postnatal lethality, making this method less than ideal for revealing the precise roles of these ligands and receptors in gonadal function.

### IGFs, sex determination and testis development

Sex determination and the sex-specific differentiation of bipotential gonads require distinct male and female genetic programs. This also involves the interdependent assembly of several somatic cell types, together with GCs, which requires a coordinated signaling program resulting in the development of either testes or ovaries. By embryonic day (E) 10.5 in mice, bipotential gonads arise from the genital ridges and are composed of primordial germ cells and steroidogenic factor 1-positive (SF1^+^) somatic cells, which include the supporting and steroidogenic cell precursors. In XY individuals, sex determination then begins with the transient expression of the Sex-determining Region of the Y chromosome (*Sry*), which in concert with SF1, triggers *Sox9* upregulation and the differentiation of supporting cell precursors into SCs [51–54]. SCs subsequently act as organizing centers to enclose GCs, form testis cords and orchestrate the differentiation of all other cell types including fetal LCs, prospematogonia and peritubular myoid cells [55–60]. In the absence of *Sry* expression, a robust female-specific genetic program is initiated in XX gonads leading to ovarian differentiation.

In recent years, increasing evidence has emerged that the insulin family of growth factors plays an essential role in gonad development and sex determination. In particular, insulin/IGF signaling is absolutely required for sex determination and testis differentiation in mice [7, 9]. XY mice that are mutant for both *Insr* and *Igf1r* develop ovaries and exhibit a completely female phenotype. Further studies have shown that mouse embryos lacking both *Insr* and *Igf1r* failed to develop testes due to a reduction in *Sf1* gene expression and a failure of *Sry* upregulation. Moreover, they exhibited reduced proliferation rates of somatic progenitor cells in both XX and XY gonads prior to sex determination [9]. In addition, the drastic reduction and delay in *Sry* expression in *Insr;Igf1r* double KO animals was correlated with the lack of upregulation of key testis genes such as *Sox9*, *Fgf9*, and *Ptgds*, and the absence of SCs, LCs and overall testis formation. Interestingly, while a few SOX9^+^ cells were found in E12.5 XY double KO gonads, these were absent at later stages, suggesting that in addition to *Sox9* activation, maintenance of *Sox9* expression was also impaired in mutant XY gonads. Recent studies have demonstrated that the first event triggered by the onset of SRY expression is the immediate accumulation of glycogen within SC precursors [61]. This energy storage seems to be critical, as disruption of glycogen synthesis and accumulation results in the failure of *Sox9* upregulation, testis cord formation and overall testis development. Interestingly, glycogen storage within pre-SCs appears to be dependent on the activation of the PI3K pathway, which is known to be activated by both insulin and IGFs [11]. In double KO *Insr-Igf1r* null embryos, there is also a delay in ovarian differentiation, which suggests that regardless of the genetic sex, gonads lacking insulin signaling remain in an undifferentiated state with no clear activation of either testicular or ovarian pathways for several days. Expression analyses during sex determination revealed that significant fractions of both the testicular and ovarian pathways were prematurely altered, which may explain the incapacity of mutant gonads to develop into either ovaries or testes at the normal time of sex determination [9]. These studies showed for the first time that, should a gonad remain uncommitted at the normal time of sex determination, it remains in an undifferentiated state for several days until the ovarian differentiation program finally takes over at E16.5.

### IGFs are major factors regulating SC number, testis size and FSH action

SCs are the only somatic cells of the seminiferous epithelium and are mainly committed to sustaining spermatogenesis. As a result of this essential role, testis size and sperm production are directly correlated to the total number of adult SCs [62]. Although several local growth factors and hormones have been reported to influence the number of SCs, such as follicle-stimulating hormone (FSH) [62–65] thyroid hormone, activin, fibroblast growth factor, epidermal growth factor, transforming growth factor α, and glial cell line-derived neurotropic factor (GDNF) [12, 66–72], a recent report revealed that IGFs are the most important growth factor in regulating SC number and testis size. Pitetti et al. [10], utilizing a conditional KO approach, investigated whether the IGF system is required for SC and GC development and function. When both *Insr* and *Igf1r* were inactivated specifically in SCs there was a 72% reduction in testis size and a 79% reduction in daily sperm production in adult testes. This resulted from the reduced proliferation of immature SCs during late fetal and early neonatal development. This reduction in total SC number and testis size was more severe than in single mutants, revealing the concerted action of both INSR and IGF1R in testicular development. Although *Insr*, *Igf1r*, and *Insr;Igf1r* mutant mice all exhibited reduced SC number and testis size, and drastic declines in sperm production, they were fertile, indicating that the absence of IGF signaling in SCs does not impair spermatogenesis directly [10]. An improved understanding of the mediation of SC number and testis size by insulin/IGF signaling pathways was provided by animal studies of IRS proteins. Mice with a complete absence of *Irs2* show a 45% reduction in testis weight, with significantly fewer SCs, spermatogonia, spermatocytes, elongated spermatids, and spermatozoa, whereas testicular development in *Irs1*-deficient mice is not altered [8]. These data indicate that IRS2 plays a critical role in testicular development, potentially by mediating IGF signaling during embryonic and early postnatal development. Nevertheless, as is the case with constitutive knockout models, the specific effects of IRS1 or IRS2 on SC development cannot be confirmed in this model due to their extensive roles in other tissues. Nevertheless, taken together, results from recent studies underscore the indispensable role that the insulin/IGF signaling pathway plays in the regulation of SC number, testis size, and daily sperm output.

Interestingly, insulin and IGF receptors also play a crucial role in mediating FSH action. FSH is considered one of the major endocrine hormones that regulate SC proliferation during testicular development [62–64]. Although FSH acts through a G protein–coupled receptor, its actions are intricately interconnected with IGF pathways, potentially through common downstream signaling pathways or via FSH-dependent secretion of IGFs. In fact, FSH amplifies IGF1-mediated activation of AKT signaling by SCs [73–76]. In mice lacking the FSH receptor specifically in SCs, testis weight and SC number were reduced by approximately 55-60% [65], compared with >70% for SC-*Insr*;*Igf1r* mice [10]. *In vivo* experiments have shown that the neonatal action of FSH requires the insulin/IGF signaling pathway to mediate its proliferative effects on immature SCs [10]. Interactions akin to those observed in the testis between the IGF system and FSH have also been reported in the ovary. In rat granulosa cells treated with IGF1, *Fshr* expression and mRNA stability were increased [77]. Furthermore, FSH-induced phosphorylation of AKT and stimulation of steroidogenic genes and estradiol production in these cells requires IGF1R activation [78]. Collectively, these results from both granulosa cells and SCs suggest a crucial role for IGFs in mediating FSH action in the gonads. These data also reinforce the view that the local production of insulin/IGFs is the major intratesticular signal regulating SC number, testis size and sperm output in mammals. In addition, although several factors play a role in the proliferation and maturation of SCs [79], insulin/IGF signaling is likely to represent the major hormonal signal involved in the establishment of a normal number of SCs.

### IGFs are dispensable for differentiating GCs during spermatogenesis

Spermatogenesis is a highly complex process in which diploid spermatogonial stem cells differentiate into mature haploid spermatozoa. Multiple genetic, hormonal, and growth factor pathways must act in concert to continually produce mature sperm. It remains unclear whether IGFs act directly on the GC lineage to regulate their survival and differentiation into mature haploid spermatozoa. IGF1 and IGF1R are expressed in spermatogonia and spermatocytes [80], implying a role in spermatogenesis as potential autocrine or paracrine factors, although little is known about their specific functions. When either *Insr*, *Igf1r*, or both receptors together are inactivated specifically in the GC lineage, GC development and spermatogenesis are unaltered and males are viable and fertile [10]. Furthermore, all reproductive parameters are normal including testicular histology, testis size, and sperm production, indicating that insulin/IGF signaling is expendable in GCs throughout the meiotic and postmeiotic stages of spermatogenesis. In contrast, the robust expression of *Igf1* and *Igf2* in male GCs [10], in particular spermatogonia, suggest that GCs represent an important source of testicular IGFs which may act in a paracrine manner to regulate different aspects of SC, LC or peritubular myoid cell, biology during testis development and later in adult life. Thus, the possibility remains that IGFs may play a role or even be required for the proliferation of gonocytes/spermatogonia. In fact, data from an earlier study suggest that IGF1 and IGF2 have a selective paracrine or autocrine role in the regulation of spermatogonial proliferation during spermatogenesis [81].

### IGFs mediate proliferation of fetal LCs and steroidogenic function of adult LCs

In mammals, two distinct populations of LCs appear during testis development: the fetal Leydig cells (FLCs) and adult Leydig cells (ALCs) [82]. FLCs produce both androgens and INSL3, which are responsible for the masculinization of the urogenital system and testicular descent (for a review see [83]). These cells regress thereafter, and are replaced by the ALC population, which appears during puberty. Currently, two major hypotheses prevail in terms of FLC and ALC development and differentiation. The first is that FLCs and ALCs originate from the same progenitor population but some of these progenitors remain dormant up until prepubertal development when they develop into ALCs [84, 85]. The second hypothesis maintains that FLCs and ALCs originate from distinct cell lineages with different origins and function [86–88].

Several factors have been implicated in triggering the differentiation of progenitor cells into FLCs, such as Desert Hedgehog (DHH) [55–57, 89–92] and the platelet-derived growth factor (PDGF) family [58]. Interestingly, there are numerous reports indicating that IGF1 is also implicated in LC development and function. For example, testosterone production is reduced by more than 80% in *Igf1*-null mice, and this deficiency is associated with a significant developmental delay in LCs and altered LH-stimulated androgen secretion *in vitro* [6]. Further reductions in the expression of steroidogenic markers in the testis such as *StAR*, *Cyp11a1* and *Cyp17a1* are also observed [6, 93, 94]. Altered steroidogenic function in LCs is also indicated by increased levels of 5α-reductase, the presence of immature LCs in the adult testis, and modified ratios of 3α-androstanediol to testosterone [93]. Moreover, when GH-deficient Snell dwarf mice, which have low IGF1 levels, are treated with IGF1, LH receptors and steroidogenic responsiveness is augmented, indicating that IGF1 induces the maturation of LC function [95]. Consistent with these findings, the reduction in ALC number in mice mutant for *Igf1* [6, 96] is a consequence of altered proliferation and differentiation of progenitor LCs between postnatal days 14 and 35, although the developmentally earlier LC stem cell lineage is not affected [93]. Furthermore, IGF1 has been shown to stimulate the proliferation of progenitor LCs [97]. Overall, these data indicate that the IGF system plays an important role not only in mediating the proliferation of precursor LCs and the establishment of a normal number of ALCs, but also in the steroidogenic capacity of ALCs.

### Conclusions, perspectives and unanswered questions

Recent studies have unequivocally demonstrated the essential role of the insulin family of growth factors in sex determination and reproductive function. Specifically, insulin/IGF signaling is absolutely required for testis differentiation, and embryos lacking both *Insr* and *Igf1r* fail to develop testes [7, 9]. The local production of IGFs appears to be the major intratesticular signal controlling SC number, testis size, and sperm output in mammals, although insulin/IGF signaling appears not to be required in GCs. In addition, IGF1 plays an essential role in the proliferation of precursor LCs, the establishment of a normal number of ALCs, and proper steroidogenesis.

Although significant progress has been made with regard to the role of the insulin family of growth factors in reproductive development and function, many critical questions remain unresolved. The first question pertains to the specific source(s) of intratesticular IGFs. Multiple cell types in the body are capable of synthesizing IGFs and multiple cell types in the testis have been implicated, although conclusive data on which are responsible for local IGF production and secretion are still lacking. The next obvious unanswered question is the precise identity of the IGFs that are produced and secreted in the testis. In general, IGF1 is normally associated with adult functions while IGF2 is known to act during embryonic development. It is not yet clear whether this general pattern holds true for testis development and function. The third area of investigation that needs further exploration is determining the relative contributions of endocrine, paracrine and autocrine effects of the IGFs in the testis. A fourth area for further study is the precise mechanisms by which IGF signaling in the testis regulates testicular development and function. For example, the actions of the insulin/IGF family of growth factors are mediated primarily through INSR and IGF1R and activate two major signaling pathways, the MAPK and PI3K pathway. FSHR activation also stimulates these two pathways, suggesting that IGF and FSH actions may be coordinated in SCs. Although INSR and IGF1R are required for FSH-mediated SC proliferation [10], the mechanism controlling these concerted actions in immature SCs is not completely understood. Lastly, the precise *in vivo* role of IGFs in LC differentiation and steroidogenesis remains to be unraveled. Data from the most recent studies have employed constitutive *Igf1*-deficient mice, making it extremely difficult to pinpoint local testis-specific mechanisms in such a noisy background. Again, the endocrine, paracrine and autocrine actions of IGFs represent a recurring and important theme in deciphering the roles of IGFs in testicular development and function. Continued technological advances in genomics, proteomics, and the availability of animal models with cell-specific KOs of particular ligands or receptors will surely aid in the quest to understand the precise roles that the insulin family of growth factors plays in reproductive development and function.

## Abbreviations

IGF: Insulin-like growth factor
SC: Sertoli cell
GC: Germ cell
LC: Leydig cell
INSR: Insulin receptor
IGF1R: Type-I insulin-like growth factor receptor
IGFBP: Insulin-like growth factor binding protein
M6P: Mannose-6-phosphate
IRS: Insulin receptor substrate
PI3K: Phosphatidyl-inositol 3-kinase
MAPK: Mitogen activated protein kinase
JAK: Janus kinase
STAT: Signal transducer and activator of transcription
SF1: Steroidogenic factor 1
SRY: Sex-determining region of the Y chromosome
SOX9: Sex-determining region of the Y chromosome box 9
FGF: Fibroblast growth factor
PTGDS: Prostaglandin D2 synthase
KO: Knockout
FSH: Follicle-stimulating hormone
GDNF: Glial cell-line derived neurotropic factor
AKT: Protein kinase B
mRNA: messenger ribonucleic acid
FLC: Fetal Leydig cell
ALC: Adult Leydig cell
DHH: Desert hedgehog
PDGF: Platelet-derived growth factor
StAR: Steroidogenic acute regulatory protein
Cyp11a1: Cholesterol side chain cleavage enzyme
Cyp17a1: 17 alpha-hydroxylase/17,20 lyase
GH: Growth hormone.
